# The Impact of Mercury Selection and Conjugative Genetic Elements on Community Structure and Resistance Gene Transfer

**DOI:** 10.3389/fmicb.2020.01846

**Published:** 2020-08-05

**Authors:** James P. J. Hall, Ellie Harrison, Katariina Pärnänen, Marko Virta, Michael A. Brockhurst

**Affiliations:** ^1^Department of Evolution, Ecology and Behaviour, Institute of Integrative Biology, The University of Liverpool, Liverpool, United Kingdom; ^2^Department of Animal and Plant Sciences, University of Sheffield, Sheffield, United Kingdom; ^3^Department of Biology, University of York, York, United Kingdom; ^4^Department of Microbiology, University of Helsinki, Helsinki, Finland; ^5^Division of Evolution and Genomic Sciences, School of Biological Sciences, The University of Manchester, Manchester, United Kingdom

**Keywords:** horizontal gene transfer, conjugative plasmids, mobile genetic elements, *Pseudomonas*, mercury, soil, bacterial communities

## Abstract

Carriage of resistance genes can underpin bacterial survival, and by spreading these genes between species, mobile genetic elements (MGEs) can potentially protect diversity within microbial communities. The spread of MGEs could be affected by environmental factors such as selection for resistance, and biological factors such as plasmid host range, with consequences for individual species and for community structure. Here we cultured a focal bacterial strain, *Pseudomonas fluorescens* SBW25, embedded within a soil microbial community, with and without mercury selection, and with and without mercury resistance plasmids (pQBR57 or pQBR103), to investigate the effects of selection and resistance gene introduction on (1) the focal species; (2) the community as a whole; (3) the spread of the introduced *mer* resistance operon. We found that *P. fluorescens* SBW25 only escaped competitive exclusion by other members of community under mercury selection, even when it did not begin with a mercury resistance plasmid, due to its propensity to acquire resistance from the community by horizontal gene transfer. Mercury pollution had a significant effect on community structure, decreasing alpha diversity within communities while increasing beta diversity between communities, a pattern that was not affected by the introduction of mercury resistance plasmids by *P. fluorescens* SBW25. Nevertheless, the introduced *merA* gene spread to a phylogenetically diverse set of recipients over the 5 weeks of the experiment, as assessed by epicPCR. Our data demonstrates how the effects of MGEs can be experimentally assessed for individual lineages, the wider community, and for the spread of adaptive traits.

## Introduction

Many of the traits that make bacteria economically, ecologically, or clinically important are encoded by accessory genes carried by mobile genetic elements (MGEs) (Hall et al., 2017a). Conjugative MGEs, i.e., those with genes that produce a channel (the conjugative pilus) through which the MGE can be copied between neighboring bacteria (Garcillán-Barcia and de la Cruz, 2013; Cury et al., 2017), are particularly important for the spread of traits in bacterial communities. This is because of the efficiency with which conjugative MGEs can transmit large accessory gene cargos between individuals, including those of different species (Halary et al., 2010; Klümper et al., 2015). By enabling adaptive traits to move into new lineages, conjugative MGEs can drive rapid evolution, and adaptation to environmental change (Hall et al., 2017a).

The impacts of MGE acquisition for adaptation can be seen at the level of an individual bacterial lineage, where trait acquisition can allow survival in the face of a new abiotic stress like disinfectants or toxic metals (Silver and Misra, 1988; Wassenaar et al., 2015), provide genes to outcompete rivals (Riley and Wertz, 2002), or enable that lineage to occupy a new niche, such as a new animal or plant host (Faruque and Mekalanos, 2003; Platt et al., 2012). Horizontal gene transfer through MGE exchange also has effects that manifest at the level of the wider bacterial community. From a community perspective, adaptive traits spread by MGEs can potentially sustain community-wide diversity — and community function — in the face of strong selection for that trait. In mouse gut microbial communities, for example, antibiotic treatment caused increased mobilization of resistance genes by bacteriophage (Modi et al., 2013), which could mediate functional resilience of the microbiome. The effects of MGE transmission can also be considered from the perspective of the trait in question. Mobile traits are likely to be more persistent relative to traits that are more tightly linked to a particular lineage, particularly at times where positive selection is weak or absent, because mobile traits can move into lineages that are better adapted to the prevailing local conditions (Bergstrom et al., 2000; Niehus et al., 2015). Probiotic treatments, designed to introduce new traits such as phytoprotection or detoxification of pollutants into microbial communities (also known as “bioaugmentation”), could therefore benefit from a consideration of the mobility of the genes encoding the introduced function (Garbisu et al., 2017).

The maintenance and spread of MGEs in a bacterial community is affected by several factors. MGE acquisition varies across taxa, and across different strains of the same species (McNally et al., 2016; Wyres and Holt, 2018). Lineages vary in their ability to acquire and maintain plasmids, due to conflicting genes such as restriction-modification systems and CRISPR immunity (Oliveira et al., 2016; Westra et al., 2016). Lineages that are favorable to MGE acquisition would therefore be predicted to be susceptible to infectious parasites like bacteriophage, but also more resilient to environmental change as they can acquire adaptive MGEs (Jiang et al., 2013; Bellanger et al., 2014; Westra et al., 2016). Patterns of MGE transmission also vary with the MGEs themselves: different MGEs vary in their host range (Jain and Srivastava, 2013; Cury et al., 2018), impose varying burdens on recipient fitness, and have differing baseline rates of transmission (e.g., Hall et al., 2015). The prevailing environmental conditions will also affect the spread of MGE-borne traits. Selection for the traits carried by MGEs can favor MGE spread by enhancing the fitness of recipients, but may at the same time reduce MGE spread by removing potential recipients from the community (Lopatkin et al., 2016; Stevenson et al., 2017). Highly transmissible MGEs can effectively spread traits in the absence of selection, particularly when MGE persistence depends on infectious transmission (Lopatkin et al., 2016; Hall et al., 2016). Although the factors driving MGE spread have been investigated in laboratory studies, there is a general lack of experimental data describing MGE transmission in the context of species-rich bacterial communities in their natural habitat, and how patterns of MGE exchange are affected by selection.

To understand how both genetic and ecological factors drive the spread of MGEs, and what the consequences are for individual lineages and the broader bacterial community, we established an experiment in which a trait was introduced into a diverse bacterial community on different conjugative plasmids, with and without positive selection for the trait. We used the *Pseudomonas fluorescens* SBW25/pQBR plasmid system. *P. fluorescens* SBW25 is a plasmid-free strain isolated from the same site as the pQBR plasmids, and thus represents a naturally relevant host. *P. fluorescens* SBW25 is plant-associated, but can proliferate in bulk potting soil, and has been studied in soil microcosm experiments both by itself and alongside the resident soil community (Lilley and Bailey, 1997b; Gómez et al., 2016; Hall et al., 2016). The pQBR plasmids were isolated by their ability to mobilize mercury resistance (Lilley et al., 1996). Though all pQBR plasmids sequenced to date contain the same mercury resistance operon located on a Tn5042 transposon, the plasmid backbones can be very different. Previous work has shown that pQBR103 and pQBR57 — conjugation-proficient megaplasmids of 425 and 307 kb, respectively — carry identical *merA* genes but pQBR103 has a larger fitness cost and a lower conjugation rate than pQBR57, when tested in *P. fluorescens* SBW25 (Hall et al., 2015). Both plasmids are known to transfer into other species of *Pseudomonas*, but their broader ranges are unknown (Hall et al., 2016; Kottara et al., 2018). Both plasmids are predominantly comprised of uncharacterized genes with unknown relevance to the soil environment, but there is evidence that some pQBR103 genes are associated with plant interactions (Lilley and Bailey, 1997a; Zhang et al., 2004). The microbial community was derived from a suspension of the same soil used in the experiments: it represents a species-rich natural assemblage likely to contain archaea and eukaryotes alongside bacteria. Though this community has been artificially extracted from potting soil by a soil wash process (which may have failed to sample some members of the original assemblage) it remains directly relevant to the experimental conditions under investigation.

We cultured *P. fluorescens* SBW25 (the “focal strain”), carrying either of two mercury resistance plasmids, pQBR57 and pQBR103, or no plasmid, and either by itself, or embedded within this semi-natural community from potting soil. These soil microcosms contained either unsupplemented potting soil or potting soil supplemented with two different concentrations of ionic mercury, in a fully factorial design. The levels of mercury used represented moderate-high, and very high levels of pollution seen in natural sites (Arbestain et al., 2008). Over the course of five growth cycles in soil microcosms, we tracked the dynamics of the focal strain, the composition of the bacterial fraction of the community as a whole, and the spread of mercury resistance.

## Materials and Methods

### Bacterial Soil Culture

*Pseudomonas fluorescens* SBW25 was previously labeled with a streptomycin resistance cassette and the *lacZ* marker gene and used as a recipient for conjugation of plasmids pQBR103 and pQBR57 (Hall et al., 2015). Strains were streaked onto Kings B media (20 g proteose peptone, 1.5 g MgSO_4_⋅7H_2_O, 1.5 g K_2_HPO_4_⋅3H_2_O, 10 g glycerol per liter, supplemented with 12 g/L agar) containing 200 μg/ml streptomycin, and 20 mM HgCl_2_ where appropriate, and isolated colonies used to set up liquid KB cultures for the experiment (one colony per replicate). Cultures were grown for 40 h to reach saturation before beginning the experiment. Soil cultures were maintained in twice-autoclaved “potting soil microcosms,” which consisted of 10 g John Innes #2 potting compost in a 30 ml glass universal tube. Before inoculation, microcosms were amended by addition of 900 μl of either water, or HgCl_2_ solution to adjust Hg^2 +^ concentration to 16 or 64 μg/g, and vortexed briefly. Microcosms were incubated at room temperature for approximately 1 h after amendment before use. Soil water content was approximately 25% v/w (Hall et al., 2015). To establish the experiment, the natural community was first extracted using a soil wash. Unautoclaved soil (200 g), from the same bag as that used to make the microcosms, was added to a 500 ml duran flask with 400 glass beads (5 mm) and 200 ml sterile M9 buffer (47.8 mM Na_2_HPO_4_, 22 mM KH_2_PO_4_, 8.55 mM NaCl, 18.7 mM NH_4_Cl, pH 7.4) and mixed thoroughly by shaking and vortexing for 5 min. Supernatant was removed into a sterile falcon tube, and sample of this was autoclaved for the “no natural community” treatments. *P. fluorescens* cultures were pelleted and resuspended in M9 buffer at 1:20 dilution. Samples were mixed 1:1 v/v with either natural community or autoclaved natural community, and 200 μl was added to the soil microcosm and vortexed briefly to disperse. Soil cultures were maintained at 28°C and 80% relative humidity.

Every 7 days, samples of soil wash from each population was transferred into fresh media. M9 buffer (10 ml) and twenty 5 mm glass beads were added to each microcosm and vortexed for 1 min. A sample of soil wash (100 μl) was transferred into a fresh microcosm to continue the experiment, and samples were spread on media to establish population densities. Routinely, samples were spread on KB agar supplemented with 50 μg/ml X-gal and 200 μg/ml streptomycin to enumerate *P. fluorescens* SBW25 cfu/g, onto 0.1× nutrient agar (NA, Oxoid) supplemented with 50 μg/ml X-gal to enumerate the total community, and onto 0.1× NA with 50 μg/ml X-gal and 2 μM HgCl_2_ to enumerate the mercury resistant portion of the natural community. Natural community plates were counted after 4 days growth at 28°C. Mercury resistance amongst *P. fluorescens* SBW25 was tracked by plating samples of culture onto KB + 200 μg/ml streptomycin + 20 μM HgCl_2_, or by replica plating from the KB + 200 μg/ml streptomycin plates onto 100 μM HgCl_2_. In some cases (e.g., from the plasmid-free populations) mercury resistance was also estimated by spreading samples on KB supplemented with 200 μg/ml streptomycin and 20 μM HgCl2. Mercury concentrations were adjusted across media types to be selective for resistance, based on results from preliminary experiments. Colony PCR was performed on up to 12 mercury-resistant endpoint clones from each surviving population to test for the presence of plasmid backbone genes (*oriV*, *trfA*) as described previously (Harrison et al., 2015; Hall et al., 2016); plasmid loss with *merA* maintenance was found in only two populations: pQBR57 with 64 μg/g Hg^2+^ with natural community, replicate a; and pQBR103 with 16 μg/g Hg^2+^ with natural community replicate d. In each case, 3/12 (25%) of tested clones had lost the pQBR plasmid but maintained *merA*.

Samples of communities for downstream analyses (16S sequencing, epicPCR) were frozen by adding glycerol to soil wash at 20% w/v final concentration and freezing at −80°C.

### Extracting Bacteria From Soil

We adapted a nycodenz centrifugation protocol from Burmølle et al. (2003) to extract bacteria from soil for 16S and epicPCR analysis. Frozen soil wash/glycerol samples were thawed and pelleted at 5 G for 5 min, and resuspended in 600 μl 0.2% w/v sodium pyrophosphate. Resuspended samples were vortexed for 1 min, then 300 μl of Nycodenz cushion (1.3 g/ml) was carefully pipetted below each sample, avoiding mixing. Samples were centrifuged for 10 min at 10.9 G, before the top layer and interface (∼700 μl) was carefully removed and added to a new tube containing 400 μl 0.85% NaCl. Samples were pelleted again at 5 G for 5 min and resuspended in 1 ml nuclease-free water. Preliminary experiments showed that this protocol often resulted in aggregates. To remove these and generate the single-cell suspension necessary for epicPCR, all samples were gently pipetted and then filtered through a 5 μm syringe filter, pelleted, and resuspended in H_2_O. A sample was taken for epicPCR bead prep and the remainder was frozen in 20% glycerol in M9 for subsequent 16S amplicon PCR.

### Generating Acrylamide Beads for epicPCR and Generation of epicPCR Amplicons

Un-lysed cells were used to generate acrylamide beads for epicPCR according to Spencer et al. (2016) with a lysozyme step for cell lysis. Full details are provided in Supplementary Methods. Samples of beads were stained with SYBR green (1:10,000) and imaged using a fluorescence microscope to ensure that >99% of beads were empty before generating emulsions for epicPCR. Beads were used as templates in the first-round of epicPCR using the primers merA_F1B, merA_F2 + R1, and R1 (Supplementary Table 7), and samples of the PCR reaction were imaged to ensure emulsion stability and the presence of only one acrylamide bead per drop. Second-round epicPCR products were generated using primers merA_F3E and PE16S_V4_E786_R. Blocking primers R1 + F1block10F and R1 + F1block10R were added to block amplification of unfused products. Quadruplicate reactions were performed for each sample and the products pooled and purified using AMPure XP beads.

### DNA Extraction for 16S Amplicon Generation

Total DNA from cells extracted using the nycodenz protocol was extracted using the DNeasy Blood & Tissue Kits’ (QIAGEN) and 5 μl used for PCR using primers PE16S_V4_U515_F and PE16S_V4_E786_R using Phusion Hot-Start Flex polymerase. Full details are provided as Supplementary Methods. Quadruplicate reactions were performed for each sample and the products pooled. 16S and epicPCR amplicons were barcoded and pooled and each library was sequenced using a MiSeq v2 with 250 bp paired-end reads. The 16S amplicon analyses generated >50,000 read pairs per sample library. Yield from epicPCR was variable due to low input from some samples.

### Community Sequence Analysis

Amplicon data was analyzed using QIIME2 (version qiime2-2018.11) (Bolyen et al., 2019) using the dada2 denoising module, and R (R Foundation for Statistical Computing, Vienna, Austria). Short read sequences can be found at the short read archive PRJEB34647.

For the 16S data, primer sequences were removed using “–p-trim-left-f 23” and “–p-trim-left-r 20” functions. Reads were truncated to maintain read quality above a PHRED-scaled score of 30, which resulted in a truncation length of 210 in the forward read and 190 in the reverse read. About 25% of reads were lost, primarily through the removal of chimeras. Low abundance sequence variants (total frequency <0.001%) were removed, leaving 4,863,898 sequences comprising 613 variants across the evolved populations. Preliminary data exploration revealed that one sample (replicate a, plasmid-free, no mercury) had a very divergent population structure which could be traced to a technical issue with DNA extraction, so this sample was excluded from the analysis. Data were subsampled to 50,000 reads for all analyses. Alpha diversity metrics were analyzed using linear models with plasmid, mercury, and their interaction as fixed effects, using Type II Sums of Squares to assess main effects, and the sjstats package (Lüdecke, 2020) was used to calculate eta-squared. Beta-diversity was analyzed by permutational MANOVA using the adonis2 function in the vegan package (Okansen et al., 2019).^1^ Dispersion for each distance measure was extracted using the betadisper function in the vegan package and analyzed as with alpha diversity. We identified a generally good correlation between plate counts for *P. fluorescens* SBW25 and abundance of reads matching the expected SBW25 amplicon sequence variant (ASV; Spearman’s rho = 0.879, *p* < 0.001). Dominant, abundant amplicon sequences can cause technical artifacts with 16S amplicon analyses. Though the SBW25 amplicon was not overwhelmingly abundant, we repeated all of the analyses with the SBW25 amplicon excluded, and found that this had no qualitative effect on our conclusions. To investigate enrichment of specific taxa across treatments we performed differential abundance analysis using balances via gneiss, implemented in QIIME2. Balances were identified that were associated with increasing and decreasing abundance with mercury, and the distributions of taxa across these balances (from phylum to genus) were tested with Chi-squared goodness-of-fit tests, with Benjamini–Hochberg correction for multiple testing.

For the epicPCR data, a preliminary analysis was first conducted to test that primers were amplifying the correct *merA* allele. Primer sequences were removed using “–p-trim-left-f 21” and “–p-trim-left-r 20” functions, and as products were expected to be fused amplicons, the data were initially denoised for preliminary analysis without chimera checking using the option “–p-chimera-method none.” Reads were truncated to 205 bp in the forward read and 180 bp in the reverse read to ensure PHRED-scaled quality scores >30. Representative sequences were analyzed for the presence of the expected *merA* fragment. Of 10,906 sequences, 8,994 contained the correct sequence for *merA*. Of the remaining sequences, approximately half were truncated 16S fragments, and approximately half had only single basepair differences from the expected *merA* fragment, suggesting that these amplicon variants are likely to have a negligible effect on data interpretation. Nevertheless, all non-matching amplicons were removed from subsequent analysis. Primers and *merA* fragments were removed from matching reads, which were denoised and merged. Samples with <1,000 reads were considered amplification failures, and so only the remaining samples (*n* = 13, all of which had >150,000 reads) were used for subsequent analyses. The two negative controls (a no-sample control, and a control representing the natural community before pQBR plasmid addition) both yielded very few reads (2 and 102, respectively), almost all of which matched *P. fluorescens* SBW25 and Enterobacterales which were abundant in other samples, and thus likely represent a low level of contamination.

Plasmid recipients were analyzed by removing the ASV corresponding to *P. fluorescens* SBW25 from all samples and subsampling to the smallest sample (2,000 reads) before proceeding with taxonomy assignment. To analyze 16S data and epicPCR data together (Supplementary Figure S6), reads from the corresponding samples were processed to remove primer sequences and the *merA* fragment. The ASV corresponding to SBW25 was removed, and samples were subsampled to 2,000 reads before running the QIIME “core diversity metrics.”

### Sequencing and Analysis of the Acquired Mobile Genetic Element

**FIGURE 1:**
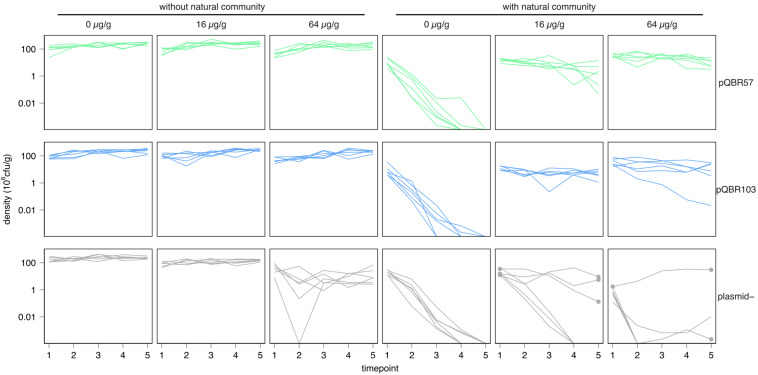
Mobile genetic elements rescued *Pseudomonas fluorescens* SBW25 in competition with a natural community in the presence of mercury stress. Each line indicates the population dynamics of *P. fluorescens* in an independent population. Different combinations of treatments are shown on separate subpanels. Subpanels are organized into rows corresponding to different *P. fluorescens* SBW25 plasmid states at the initiation of the experiment (“plasmid–” = no added plasmid), into columns corresponding to different mercury pollution treatments, and into a left and right block corresponding to absence/presence of the natural community. Lines are colored according to plasmid treatment for consistency with other figures. Timepoint indicates transfers, which occurred weekly. Dots in the mercury-treated plasmid– populations indicate populations and timepoints from which single mercury resistant clones were isolated for sequencing. Six replicate populations were established for each combination of treatments.

Nine specific mercury resistant clones, identified by growth on KB agar amended with 20 μM HgCl_2_, were selected for sequencing. These isolates represented “early” (retrieved from the first transfer) and “late” samples (retrieved at the end of the experiment) (Figure 1). Samples of bacteria were sent for short-read sequencing at MicrobesNG (Birmingham, United Kingdom). Reads were mapped to the *P. fluorescens* SBW25 chromosome (EMBL accession AM181176) using bwa-mem (Li and Durbin, 2009), and non-mapping reads were extracted using the “-f 4” option. For each sample, non-mapping reads were assembled using SPAdes (Bankevich et al., 2012), and contigs >1,000 bp extracted (the *merA* gene is approximately 1.6 kb, so this threshold was unlikely to exclude any relevant genes). All samples were found to have three contigs of similar sizes: 52, 3.3, and 2.6 kb. Corresponding contigs from each sample were aligned and examined. The 3.3 kb contigs matched the *lacZ* gene, whereas the 2.6 kb contigs carried the streptomycin 3′-adenylyltransferase gene (*aadA*). Both of these fragments were known to have been inserted into the experimental strain prior to inoculation, as resistance and reporter constructs (Zhang and Rainey, 2007; Hall et al., 2015). The 52 kb candidate was therefore the candidate mercury resistance element. Corresponding contigs from the different samples were aligned and trimmed to the same length, and were found to be identical.

Annotation of this putative mercury resistance element using the RAST server^2^ (Aziz et al., 2008) predicted a *merRTPCABD* operon, which has a *merD* gene absent from the pQBR plasmid Tn5042 *mer* operon. Additionally, the *merRTPCAB* genes were divergent from those of Tn5042, with 71.5% nucleotide identity. Specific mercury resistance had therefore been acquired independently of the pQBR plasmids. The mercury resistance element carried a Rep_3 superfamily plasmid replication initiator protein gene (ORF 21), as well as putative plasmid partitioning proteins (ORFs 12 and 23). However, an integrase was identified at the 5′ end of the sequence, and in each sample, the candidate element was identified in whole genome *de novo* assemblies, with sufficient contiguous sequence at the ends to identify a putative insertion site into the *P. fluorescens* SBW25 chromosome. Sequencing coverage across the mercury resistance element and the contiguous *P. fluorescens* SBW25 chromosome was approximately 1:1. The insertion site resulted in a 12 bp duplication at 1181688.1181699 (GAGTGGGAGTGA) on the reverse strand of the reference sequence. This region is at the 3′ end of the *guaA* gene encoding GMP synthase (PFLU_5043), a locus that is a common target for integrative and conjugative elements (ICE) (Burrus et al., 2002; Song et al., 2012). The fact that the element transferred into *P. fluorescens* SBW25, and is predicted to carry the genes required for conjugation (MOB_P__1_/MPF_T_ system identified using the MacsyFinder CONJscan module (Cury et al., 2020), also identified from RAST prediction, and by tblastx similarity to plasmid RK2) led us to consider the mercury resistance element to be an ICE. A transposon number was requested from the Tn registry (Tansirichaiya et al., 2019) and the mercury resistance element was designated integrative and conjugative element (ICE)6775. Putative CDS, identified and annotated using RAST, were supplemented with manual functional predictions based on InterProScan 5 and BLASTP queries of the NCBI refseq database, and the sequence was submitted to GenBank and given accession number MT279197.

### Statistics

Single-species *P. fluorescens* SBW25 population dynamics were analyzed using a mixed effects model in nlme with mercury and plasmid and their interactions as main effects, and a random effect of population to account for repeated measures. Dynamics of *P. fluorescens* SBW25 in the presence of the natural community were analyzed using linear models of cumulative densities across the experiment to resolve heteroscedasticity (resulting from population extinctions at later timepoints), with mercury and plasmid and their interactions as main effects. Effects of the natural community were assessed by comparing measurements at transfer 1, with mercury, plasmid, natural community, and their interactions as main effects. Effects on the natural community (both total density, and mercury resistant density) were assessed using a mixed effects model in nlme with mercury, timepoint, plasmid and their interactions as main effects, and a random effect of population to account for repeated measures. The assumptions of parametric modeling were tested using Q-Q and residual plots, Shapiro–Wilk, Fligner, and Bartlett’s tests, and data Box-Cox transformed as necessary.

### Data Availability

Short read sequencing data associated with this study can be found on the Short Read Archive (SRA) using accession PRJEB34647. The sequence of ICE6775 can be found on Genbank, accession MT279197. Other data and sample analysis scripts can be found on the University of Liverpool DataCat, doi: 10.17638/datacat.liverpool.ac.uk/1076.

## Results

### The Focal Strain: Addition of Mercury Promoted *P. fluorescens* Persistence in the Soil Microbial Community

Consistent with previous studies, *P. fluorescens* SBW25 grew well in soil microcosms when cultured alone (Figure 1 and Supplementary Figure S1, left panels). A negative effect of mercury pollution at high levels (64 μg/g) on the density of *P. fluorescens* SBW25 over time was detected in the plasmid-free treatment [linear mixed effects model (LMM), likelihood ratio test (LRT) plasmid:mercury:timepoint interaction, χ^2^ = 9.91, *p* = 0.007], but these populations persisted at levels ∼10% of those of plasmid bearers.

In contrast, *P. fluorescens* SBW25 densities were strongly suppressed when grown within the natural potting soil community, when cultured in unpolluted microcosms (linear model of densities at transfer 1, main effect of natural community *F*_1_,_90_ = 269.0, *p* < 0.0001; Figure 1 and Supplementary Figure S1, right panels). In all populations, with and without plasmids, density of *P. fluorescens* SBW25 reduced below the detection threshold (estimated as 220 cfu/g soil) over the course of the experiment, suggesting that *P. fluorescens* SBW25 was a poor competitor in the absence of mercury. It is likely that there existed one or more other members of the community that competitively excluded *P. fluorescens* SBW25 under unpolluted conditions. Mercury treatment at both moderate (16 μg/g) and high (64 μg/g) levels enhanced the persistence of both pQBR57- and pQBR103-bearing *P. fluorescens* SBW25 within the soil community (linear model of cumulative densities, plasmid:mercury interaction *F*_4_,_24_ = 13.77, *p* < 0.0001, main effect of mercury *F*_2_,_45_ = 19.5, *p* < 0.0001). Selection for plasmid-borne specific resistance genes carried by the otherwise uncompetitive *P. fluorescens* SBW25 thus apparently enhanced its competitiveness.

Surprisingly, mercury pollution also enhanced persistence of plasmid-free *P. fluorescens* SBW25 when embedded within the soil community. By the end of the experiment, 3/6 populations grown with 16 μg/g mercury, and 3/6 of those grown with 64 μg/g mercury, had detectable *P. fluorescens* SBW25, in contrast with the extinctions observed in the absence of mercury. Replica plating of samples onto mercury-supplemented media indicated that these populations of *P. fluorescens* SBW25 had acquired specific mercury resistance. No similar specific resistance was found for plasmid-free SBW25 evolved without the natural community.

**FIGURE 2:**
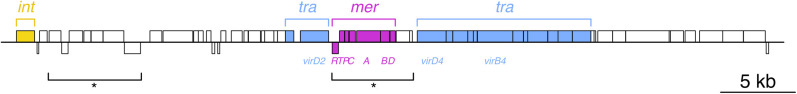
Acquisition of ICE6775 conferred specific mercury resistance to *P. fluorescens* SBW25 that did not begin with a pQBR plasmid in mercury-polluted environments. ICE6775 is 52,235 bp and carries 60 predicted coding sequences (CDS). Blocks indicate CDS, those above the line run 5′–3′ left to right across the page, whereas those below the line are 5′–3′ right to left. Key regions are indicated and colored: *int* = P4-like tyrosine recombinase; *tra* = conjugative machinery, with major components *virD2* relaxase, *virD4* coupling protein, and *virB4* major ATPase indicated below; *mer* = mercury resistance operon, with *mer* gene names indicated below. Asterisks indicate regions that were absent from the closest BLASTN hits as performed April 2020, exemplifying the mosaic nature of mobile genetic elements. In all cases, ICE6775 inserted toward the 3′ end of *guaA* GMP synthase, resulting in a 12 bp GAGTGGGAGTGA tandem duplication at each end.

Specific mercury resistance could have emerged either by *de novo* mutation or by horizontal acquisition of resistance genes from the natural community. To distinguish between these possibilities, we conducted whole genome sequencing of clones from 5 of these populations, and identified a 52 kb ICE6775 encoding mercury resistance had integrated into the *P. fluorescens* SBW25 chromosomes of all evolved clones, explaining their acquired mercury resistance (Figure 2, see section “Materials and Methods” for details). Attempts to conjugate ICE6775 from *P. fluorescens* SBW25 into a gentamicin-resistant recipient using 20 μM mercury chloride for selection did not succeed, regardless of whether mating took place in liquid KB broth or in soil microcosms. It is therefore possible that ICE6775 was mobilized by other elements into *P. fluorescens* SBW25, and/or that ICE6775 is not conjugation competent in *P. fluorescens* SBW25, at least under the tested conditions. Although we did not identify the specific member of the natural community that was the donor of this ICE, BLAST analyses identified a similar ICE present in other soil proteobacteria, including *Burkholderia*, *Pseudomonas*, and *Rahnella*. These data suggest that an environmental stress, to which *P. fluorescens* SBW25 was initially vulnerable, enabled the survival of *P. fluorescens* SBW25 in a competitive community, due to the ability of *P. fluorescens* SBW25 to acquire novel genetic material by conjugative transfer.

### The Community as a Whole: Composition Was Affected by Mercury Treatment, but Not Plasmid Addition

Mercury pollution had a significant effect on the natural community as assessed by culture on 0.1× nutrient agar (i.e., the culturable heterotrophic compartment), boosting both mercury resistance over time (LRT, mercury:timepoint, χ^2^ = 46.89, *p* = 6.6e-11), and the culturable portion of the community (LRT, effect of mercury χ^2^ = 28.05, *p* = 8.1e-07), probably through species sorting shifting the community composition toward fast-growing and thus more easily cultured taxa (Rasmussen and Sørensen, 2001; Supplementary Figure S2). We did not find support for the hypothesis that addition of the mercury resistance plasmid affected the overall success of the culturable fraction of the population under mercury pollution, indeed we found no significant effect of plasmid treatment or any higher-order interactions on either the culturable fraction of the natural community (all effects *p* > 0.11) nor on the size of the mercury resistant compartment (all effects *p* > 0.4; Supplementary Figure S2). This suggests that any effects of resistance plasmid addition were overwhelmed by pre-existing mercury resistance in the community, as exemplified by the presence of ICE6775 carrying mercury resistance (Figure 2).

**FIGURE 3:**
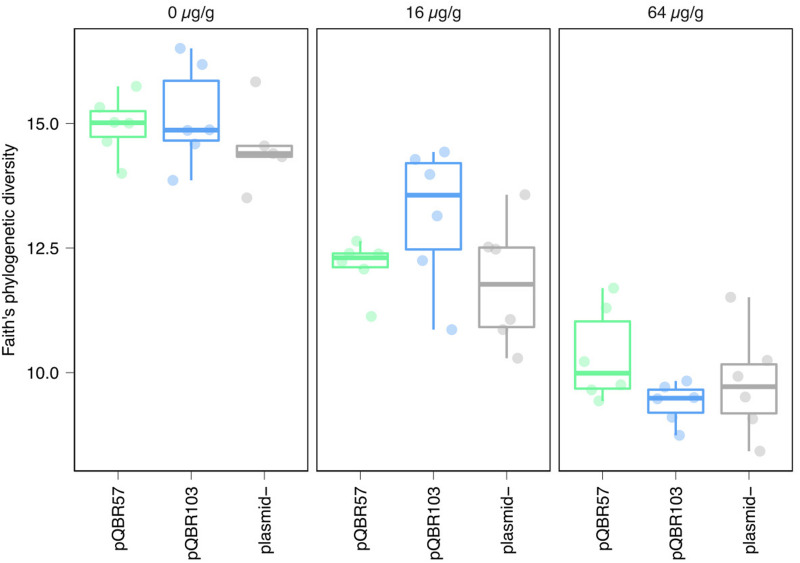
Increased mercury pollution decreased within-sample (alpha) diversity regardless of plasmid treatment. Each point indicates a population, with different colors and panels indicating the different plasmid and mercury treatments. Groups of replicate treatments are summarized with an overlaid boxplot, where the thick horizontal line indicates the median. Plots showing alternative alpha diversity metrics are provided in Supplementary Figure S3.

To understand how mercury pollution and mercury resistance plasmid addition affected the composition of the entire bacterial community, we conducted 16S amplicon sequencing on the endpoint samples. Mercury pollution reduced species richness (alpha diversity estimated by Faith’s phylogenetic divergence, *F*_2_,_48_ = 114.67, *p* < 2e-16), consistent with species sorting favoring more resistant and/or faster-growing strains (Figure 3). No significant effect of plasmid treatment, either as an interaction with mercury or as a main effect, was identified (plasmid:mercury interaction *F*_4_,_44_ = 1.96, *p* = 0.12; main effect of plasmid *F*_2_,_48_ = 1.44, *p* = 0.25). Similar trends were also noted with alternative alpha diversity measures (Pielou’s evenness, Shannon’s H, Supplementary Figure S3).

**FIGURE 4:**
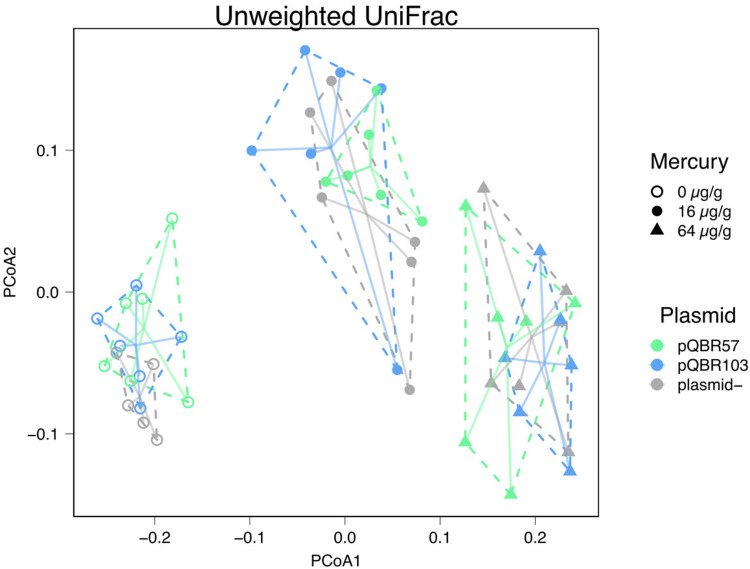
Mercury pollution shifted community composition, with effects that were not ameliorated by plasmid addition. Principal coordinates analysis of unweighted UniFrac distances. Each point indicates a population, with different colors indicating different plasmid treatments, and shapes indicating mercury treatments. Groups of replicates subjected to the same combination of treatments are enclosed within dotted lines and are connected to their group centroid with solid lines. PCoA1 = 38.6% of the variance; PCoA2 = 8.8% of variance. Plots showing analyses conducted with alternative distance measures are provided in Supplementary Figure S4.

**FIGURE 5:**
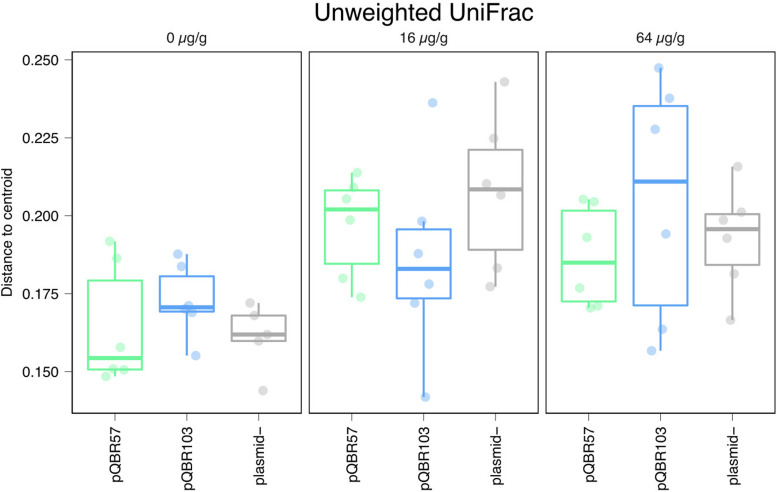
Mercury pollution increased community compositional divergence between-replicates. Distance for each population from corresponding treatment centroids, calculated from unweighted UniFrac data presented in Figure 4. Points and bars are colored as Figure 3. Plots showing alternative beta diversity metrics are provided in Supplementary Figure S5.

Alongside the negative effect that mercury had on alpha diversity, we also detected a significant effect of mercury on community composition suggesting that pollution shifted community structure in a broadly consistent manner across replicates of the same treatment, primarily through species presence/absence (Figure 4, unweighted UniFrac measure, effect of mercury, pseudo-F = 20.1, *p* = 0.001; weighted UniFrac pseudo-F = 5.13, *p* = 0.001; all effects of plasmid *p* > 0.3; Supplementary Tables 1–3). At the same time, community structure across replicate populations diverged with increasing concentrations of mercury (Figure 5, distances to centroid, effect of mercury unweighted UniFrac *F*_2_,_44_ 9.6, *p* < 0.001; weighted UniFrac *F*_2_,_44_ = 32.3, *p* < 0.001; Supplementary Tables 4–6). A significant main effect of plasmid treatment was detected only when species relative abundance was considered (weighted UniFrac *F*_2_,_44_ = 6, *p* = 0.005) but the effect was small (η^2^ = 0.097).

We detected some differences in the distribution of taxa that were enriched or depleted with increasing mercury at the Order and Family levels (Chi-squared test, p_adj_ = 0.009 for both levels). Pseudomonadales and Xanthomonadales were enriched in the pool of taxa that increased with increasing mercury, whilst Bacillales, Burkholderiales, Rhodospirillales, Sphingobacteriales were represented in the pool of taxa that were depleted as mercury concentration increased and were not amongst the taxa that were enriched.

Together, the results from 16S amplicon analyses contribute to an overall picture whereby mercury pollution generally favors a shift in population structure toward a subset of lineages, but their exact identity and relative abundance varies stochastically across replicates. Plasmid addition had a negligible effect on community composition regardless of mercury pollution.

### The Resistance Gene: Both Plasmids Mobilized Resistance to a Phylogenetically Broad Range of Recipients

Previous experiments have shown that pQBR57 and pQBR103 vary in their transmission between isogenic *P. fluorescens* SBW25 strains, suggesting that spread of the mercury resistance genes through the community may vary depending on plasmid backbone (Hall et al., 2015). To understand how the different plasmids, and application of mercury pollution, affected transmission of the introduced mercury resistance operon, we used epicPCR. epicPCR is an emulsion amplicon library preparation technique, whereby primers ensure that the V4 region of the 16S gene is amplified from single cells only when a gene of interest is present (Spencer et al., 2016). By performing the reaction on single cells trapped in “beads” of an emulsion, 16S amplicons are only generated from those individuals with the gene of interest. We designed primers targeting the specific *merA* allele introduced on pQBR103 and pQBR57 and performed epicPCR on endpoint samples to determine what members of the community had acquired mercury resistance from the introduced plasmids. Note that as our primers were designed to target a specific region of Tn5042 *merA* they would not bind the divergent ICE6775 *merA* (10/19 mismatches for the forward primer, 5/18 mismatches in the reverse primer).

We found that epicPCR consistently highlighted a subset of the community as harboring the introduced *merA* allele, that had a composition distinct from that indicated by bulk 16S amplicon sequencing (Supplementary Figure S6). After removing the original *P. fluorescens* SBW25 donor from the analysis, we found that *merA* had mostly transferred into other Gammaproteobacteria, particularly Pseudomonadales and Xanthomonadales. However, we also detected *merA* transmission to more phylogenetically distant taxa, including Burkholderiales [which often possess multireplicon genomes and thus represent potentially favorable plasmid recipients (diCenzo and Finan, 2017)], Rhizobiales, and even Bacillales. We note that these data do not necessarily imply pQBR maintenance in these recipient bacteria, since “dead-end” transmission would still yield epicPCR products. Indeed, given that the pQBR plasmids’ *merA* gene is located on an active transposon (Tn5042) (Hall et al., 2017b) it is possible that merA has translocated onto other replicons by various mechanisms, which were subsequently transferred into recipients. Nevertheless, our data is consistent with our previous findings showing pQBR103 and pQBR57 readily transmit between diverse *Pseudomonas* species (Kottara et al., 2018).

**FIGURE 6:**
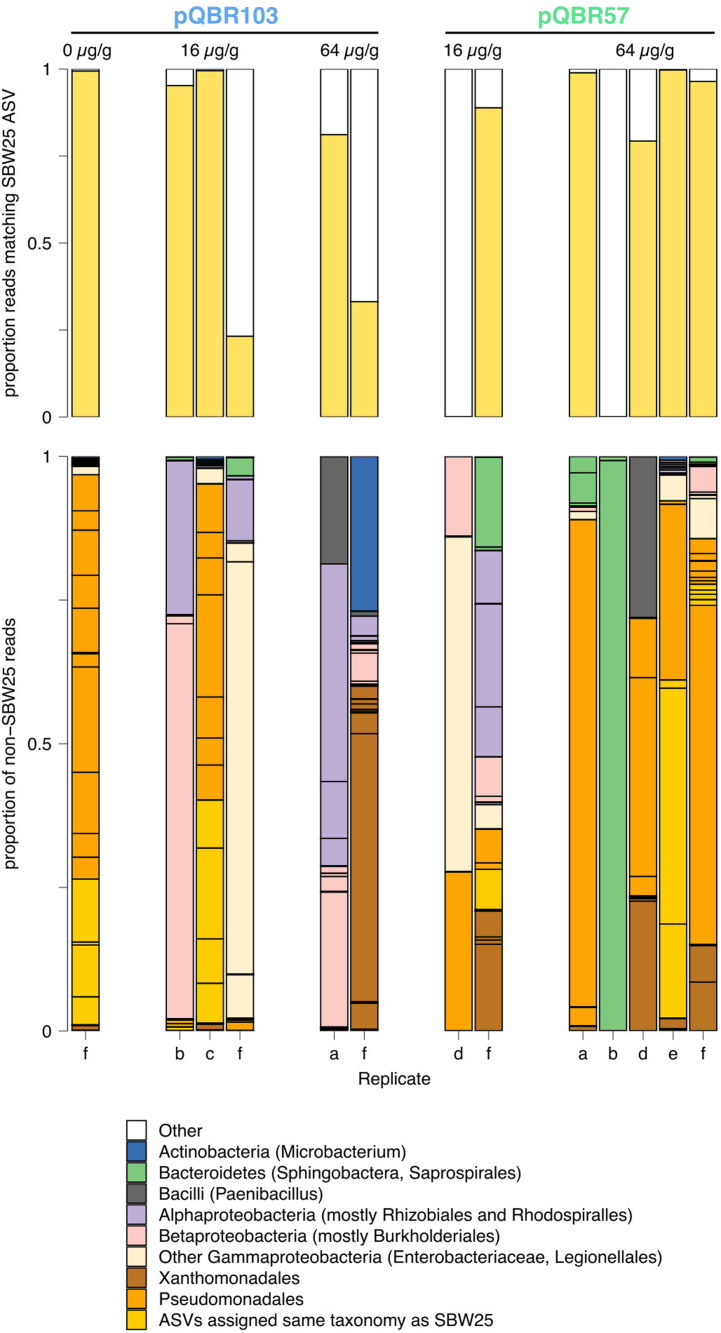
epicPCR analysis shows *merA* transmission into a diverse range of recipients in the soil community. **Top:** yellow bars indicate, for each sample, the proportion of reads from the epicPCR data that exactly match the expected 16S sequence from the *P. fluorescens* SBW25 donor. **Bottom:** bar chart showing, for each sample, the proportion of non-SBW25 reads matching different amplicon sequence variants (ASV). Black outlines indicate different ASV, colored according to broad phylogenetic category described in the legend below. Populations are grouped according to treatment.

We did not obtain sufficient epicPCR data from enough samples to statistically compare between mercury and plasmid treatments, but a visual inspection of Figure 6 and Supplementary Figure S6 do not show any obvious clustering of the different treatments. We were not able to conclude, therefore, whether mercury stress or plasmid identity had an effect on *merA* transmission into the community.

## Discussion

By taking an experimental evolution approach to study entire microbial communities, we show how community structure responds to an environmental change, in this case mercury pollution, and, furthermore, how MGEs play a critical role by transferring adaptive genes among lineages. Our data provides a clear example of how receptiveness to MGE acquisition can enhance adaptation of a bacterial lineage in a changing environment. Our focal strain, *P. fluorescens* SBW25, was uncompetitive in the presence of the natural community under normal conditions. However, a new environmental stress, mercury, promoted *P. fluorescens* SBW25 even when that strain did not originally possess mercury resistance, because *P. fluorescens* SBW25 acquired the mercury resistance element ICE6775 from the broader community. We hypothesize that *P. fluorescens* SBW25 is relatively receptive to acquisition of new MGEs, endowing it with an adaptability that underpins its success in changing environments. Indeed, previous studies have shown that *P. fluorescens* SBW25 can rapidly evolve to accommodate new conjugative plasmids, relative to other *Pseudomonas* species (Kottara et al., 2018; Hall et al., 2019), a factor that may enable this plant-associated microbe to exploit plant-associated niches during the course of the growing season (Lilley and Bailey, 1997b). That *P. fluorescens* SBW25 was competitively excluded in unpolluted environments is perhaps not surprising, because it is likely that the bacteria resident in potting soil would be better adapted to that environment than an incomer that was previously isolated from the sugar beet phyllosphere (Bailey et al., 1995). It is interesting to consider why competitive exclusion was less effective under mercury selection. Presumably, the competitor(s) in the broader community were either less able to acquire, or less able to maintain, functioning mobile mercury resistance. MGE acquisition can be impeded by various mechanisms. By inserting into a resident replicon, ICE can have a broader host range than plasmids and are not so constrained by incompatibility (Cury et al., 2018), but ICE transmission can be inhibited by resident surface- or entry-exclusion systems as well as genome defense loci such as restriction-modification or CRISPR (Brockhurst et al., 2019). Many CRISPR spacers in sequenced genomes target elements of the conjugation machinery, which acts to reduce flow of adaptive traits (Jiang et al., 2013; Westra et al., 2016; Shmakov et al., 2017). Notably, *P. fluorescens* SBW25 does not have an identified CRISPR/Cas system (Couvin et al., 2018). In addition, acquisition of resistance could have imposed lower fitness costs in *P. fluorescens* SBW25 compared with its competitor. We were not able to measure the effects of ICE6775 acquisition in our study because we could not transfer ICE6775 from *P. fluorescens* SBW25, despite a predicted functional conjugation system. Nevertheless, maintenance of acquired MGEs is known to differ between recipient genetic backgrounds, in part through varying fitness costs (De Gelder et al., 2007; Kottara et al., 2018). It would be interesting for future studies to investigate whether capacity for adaptation via MGE acquisition in the face of environmental change trades off against competitive ability under less stressful environments.

Mercury pollution reduced within-community diversity and caused the composition of the natural community to diverge between replicates (i.e., increased beta diversity). Previous studies examining the effects of environmental stressors on microbial communities have found broadly similar patterns. An investigation into the consequences for soil microbial communities of the underground passing of a coal seam fire in Centralia, Pennsylvania showed a reduction in within-community (alpha) microbial diversity driven by strong environmental filtering caused by high temperatures. Interestingly, as with mercury pollution here, the microbial communities in Centralia also underwent an increase in between-community (beta) diversity during the period of maximum stress (high soil temperatures) (Lee et al., 2017). The authors of that study suggest that the between-community variability is due to priority effects, in their case arising from the stochastic emergence of thermotolerant bacteria from dormancy. Similar patterns may be at play in our experiments, where the identity of species that come to occupy the niches rendered vacant by the inhibition of mercury-sensitive taxa is either non-deterministic, or has not been given sufficient time to equilibrate. Frossard et al. (2017) found that increasing mercury pollution in seven different natural soils shifted bacterial and fungal community composition by reducing alpha diversity, consistent with our data, though in their study soil type remained the main factor explaining community structure. Rasmussen and Sørensen (2001) found that mercury pollution of soil microbial communities had an immediate negative impact on genetic diversity, and though the overall effect weakened over time this was due predominantly to the appearance of new strains rather than the recovery of the prior community. Together this suggests that in selecting for resistant — or at least tolerant — taxa, the stress imposed by mercury decreased the diversity of communities and drove between-community differences. In our experiments, it is notable that neither of these ecological processes was significantly ameliorated by the addition of mercury resistance genes on plasmids.

Ionic mercury (i.e., Hg^2+^ such as was added to the communities in our experiments) is toxic owing to its high affinity for sulfhydryl (thiol) groups which disrupts protein function (Boyd and Barkay, 2012). The *mer* operon confers resistance because of the activity of MerA, a mercuric reductase that transfers electrons to the mercuric ion to transform it into elemental mercury (Hg^0^), a relatively unreactive gas that diffuses away (Barkay et al., 2003; Boyd and Barkay, 2012). Resistance encoded by *mer* therefore has a social aspect, in that *mer*-carrying bacteria detoxify their extracellular environment enabling otherwise susceptible bacteria to survive and proliferate (O’Brien and Buckling, 2015). We expected that introduction of pQBR mercury resistance plasmids to communities experiencing heavy mercury pollution would have affected community composition by preserving otherwise sensitive strains and increasing alpha diversity, relative to the treatments where no additional mercury resistance plasmids were added. Sensitive strains might have been protected either by acquiring *mer* by horizontal gene transfer, or as a side effect of detoxification by *mer* carried by the focal strain. However, as we did not detect a significant effect of pQBR plasmid treatment, it is likely that mercury resistance already resident in the soil wash community — in ICE6775 and probably also other instances — rendered the introduced *mer* operon redundant or diminished its effects. Mercury resistance is ubiquitous, and *mer*-harboring MGEs are diverse in natural soil communities (Lilley et al., 1996; Drønen et al., 1998; Smit et al., 1998; Sen et al., 2011), even from sites which have not experienced recent mercury pollution. The field from which the pQBR plasmids were isolated was pristine with no specific mercury pollution (Lilley et al., 1996). Indeed, though increased environmental concentration of mercury is associated with industrialization, mercury resistance MGEs have even been identified in ancient Arctic permafrost (Mindlin et al., 2005), so it is not surprising that *mer* was present in the soil wash community we isolated from unpolluted potting soil. The low fitness costs of this operon (Stevenson et al., 2017) (due to repression by MerR in the absence of mercury) and its association with diverse and efficient MGEs (Nakahara et al., 1977; Pal et al., 2015) are likely to be instrumental in the widespread presence of *mer* (Boyd and Barkay, 2012).

Resident MGEs may have been better adapted to spread in the communities than the introduced pQBR-borne resistance. Nevertheless, using epicPCR we were able to detect transmission of the introduced *merA* allele into diverse recipients. The principal recipients were other Gammaproteobacteria, particularly Pseudomonadales (most closely related to *P. fluorescens* SBW25), and Xanthomonadales (a large group of soil- and plant-associated bacteria), though we found a non-negligible subset of recipients from more phylogenetically distant taxa. Both pQBR57 and pQBR103 are known to transmit across different *Pseudomonas* species, but neither plasmid conforms to previously characterized incompatibility (Inc) groups and the extents of their host ranges are unknown. However, as we tracked the *merA* allele and not the plasmids themselves, our data describes the capacity of these plasmids to transmit a resistance gene into the community, rather than the host ranges of the plasmids *per se*. In our experiments, the *mer* operon is located on a Tn5042 transposon on the plasmids. Mercury resistance transposons like Tn5042, Tn21, and Tn5041 (Liebert et al., 1999; Kholodii et al., 2002) can efficiently transfer *mer* between conjugative elements, potentially allowing onward spread by the activity of diverse genetic vehicles. We have previously shown that Tn5042 readily transfers from the pQBR plasmids onto other replicons (Harrison et al., 2015; Hall et al., 2017b; Kottara et al., 2018), and this property may explain why we detected the introduced *merA* allele in very phylogenetically distant hosts, like *Bacillus*, that would not necessarily be expected to maintain *Pseudomonas* plasmids (Jain and Srivastava, 2013). Another possibility is that *merA* was detected from pQBR plasmids that had transferred into diverse taxa, but were not able to replicate in these recipients. Previous studies have found proteobacterial plasmid transmission to a broad phylogenetic range of bacteria, including Gram-positive recipients (Klümper et al., 2015), and even if carriage is transient within a lineage, the evolutionary and ecological consequences could be significant if accessory genes are able to relocate to the chromosome prior to plasmid loss. Future work, tracking both adaptive traits and their vehicles, will provide a detailed picture of the routes by which genes spread in complex communities, crucial to understanding how microbial communities respond to selective pressures such as antibiotic and industrial pollution (Garbisu et al., 2017; Smalla et al., 2018).

Horizontal transfer of resistance genes plays a central role in bacterial evolution and ecology even over relatively short timescales. Innovative approaches to understand HGT in experimental settings and on the scale of the microbial community, including fluorescence approaches (Klümper et al., 2016), meta-C sequencing (Stalder et al., 2019), and epicPCR (Cairns et al., 2018), represent powerful tools to survey community responses to ecological treatments, enabling experimental analyses to unpick the relative contributions of these evolutionary drivers. Tracking the patterns and consequences of HGT for individual lineages, for the genes involved, and for the structure and function of the broader microbial community will underpin the design of effective interventions to mitigate or control resistance gene spread.

## Data Availability Statement

The datasets presented in this study can be found in online repositories. The names of the repository/repositories and accession number(s) can be found at: https://www.ebi.ac.uk/ena, PRJEB34647, http://datacat.liverpool.ac.uk/1076/ and doi: 10.17638/datacat.liverpool.ac.uk/1076.

## Author Contributions

JH, EH, and MB designed the study. KP and MV developed reagents and assisted with epicPCR. JH performed the experiments and analyzed the data. JH and MB drafted the manuscript. All authors contributed to the article and approved the submitted version.

## Conflict of Interest

The authors declare that the research was conducted in the absence of any commercial or financial relationships that could be construed as a potential conflict of interest.

## Footnotes

1 https://CRAN.R-project.org/package=vegan

2 https://rast.nmpdr.org/

## Supplementary Material

FIGURE S1 Mercury resistance dynamics in *P. fluorescens* SBW25 largely mirror the broader population dynamics. Lines in black are drawn according to Figure 1. Lines in red indicate dynamics of the mercury resistant compartments of the populations. Six replicate populations were established for each combination of treatments.

FIGURE S2 Population dynamics of the total community. Lines in black describe total population dynamics, while lines in red indicate dynamics of the mercury resistant compartments of the populations, as with Figure S1. Note that population dynamics were calculated from cfu grown on 0.1× nutrient agar and thus represents only part of the culturable heterotrophic portion of the community. Six replicate populations were established for each combination of treatments.

FIGURE S3 Effects of experimental treatments on alpha diversity (Shannon’s H, Pielou’s evenness). Figures are displayed as Figure 3. We detected a significant effect of mercury, but not plasmid, on Shannon’s H (effect of mercury, *F*_2_,_48_ = 11.8, *p* = 6.63e-5; effect of plasmid *F*_2_,_48_ = 1.7, *p* = 0.19). We did not detect significant effects of either treatment on Pielou’s evenness (effect of mercury, *F*_2_,_48_ = 2.5, *p* = 0.09; effect of plasmid *F*_2_,_48_ = 1.01, *p* = 0.37).

FIGURE S4 Effects of experimental treatments on community composition differences (Bray–Curtis distance, weighted UniFrac). Principal coordinates analysis of Bray–Curtis (top) and weighted UniFrac (bottom) distances. Plot is displayed as Figure 4. For Bray–Curtis, PCoA1 = 21.6% of the variance; PCoA2 = 11.8% of variance; effect of mercury pseudo-F = 6.13, *p* = 0.001; effect of plasmid pseudo-F = 0.85, *p* = 0.64. For weighted UniFrac, PCoA1 = 42.9% of the variance; PCoA2 = 16.7% of variance; effect of mercury pseudo-F = 5.23, *p* = 0.001; effect of plasmid pseudo-F = 0.99, *p* = 0.43.

FIGURE S5 Effects of experimental treatments on community composition dispersion (Bray–Curtis distance, weighted UniFrac). Beta dispersion analysis of Bray–Curtis (top) and weighted UniFrac (bottom) distances. Plot is displayed as Figure 5. For Bray–Curtis, plasmid:mercury interaction *F*_4_,_44_ = 0.3, *p* = 0.8723. For weighted UniFrac, plasmid:mercury interaction *F*_4_,_44_ = 1.22, *p* = 0.32.

FIGURE S6 epicPCR samples a separate compartment of the community to general 16S amplicon sequencing. Principal coordinates analysis of unweighted UniFrac (top), Bray–Curtis (middle), and weighted UniFrac (bottom) distances, comparing epicPCR and whole-population 16S amplicon sequencing approaches. The amplicon corresponding to *P. fluorescens* SBW25 was removed to ensure that only the effects of *merA* transmission were analyzed. Each point indicates a sample, with colors and shapes indicating the treatment of the corresponding population (colors indicating different plasmid treatments, shapes indicating mercury treatments). Solid lines connect replicate treatments to the group centroid. Samples prepared with the same technique (epicPCR or 16S) are enclosed within dotted lines, and the area is shaded for the epicPCR samples for clarity. The variances explained by each axis for each distance are as follows: unweighted UniFrac PCoA1 = 47.2%, PCoA2 = 8.7%; Bray–Curtis PCoA1 = 16%, PCoA2 = 11.2%; Weighted UniFrac PCoA1 = 34.8%, PCoA2 = 24.3%.
