# Supplementary figures and images for: The Impact of Mercury Selection and Conjugative Genetic Elements on Community Structure and Resistance Gene Transfer
Source: Front Microbiol. 2020 Aug 5;11:1846. doi: 10.3389/fmicb.2020.01846 (PMC7419628; doi:10.3389/fmicb.2020.01846)

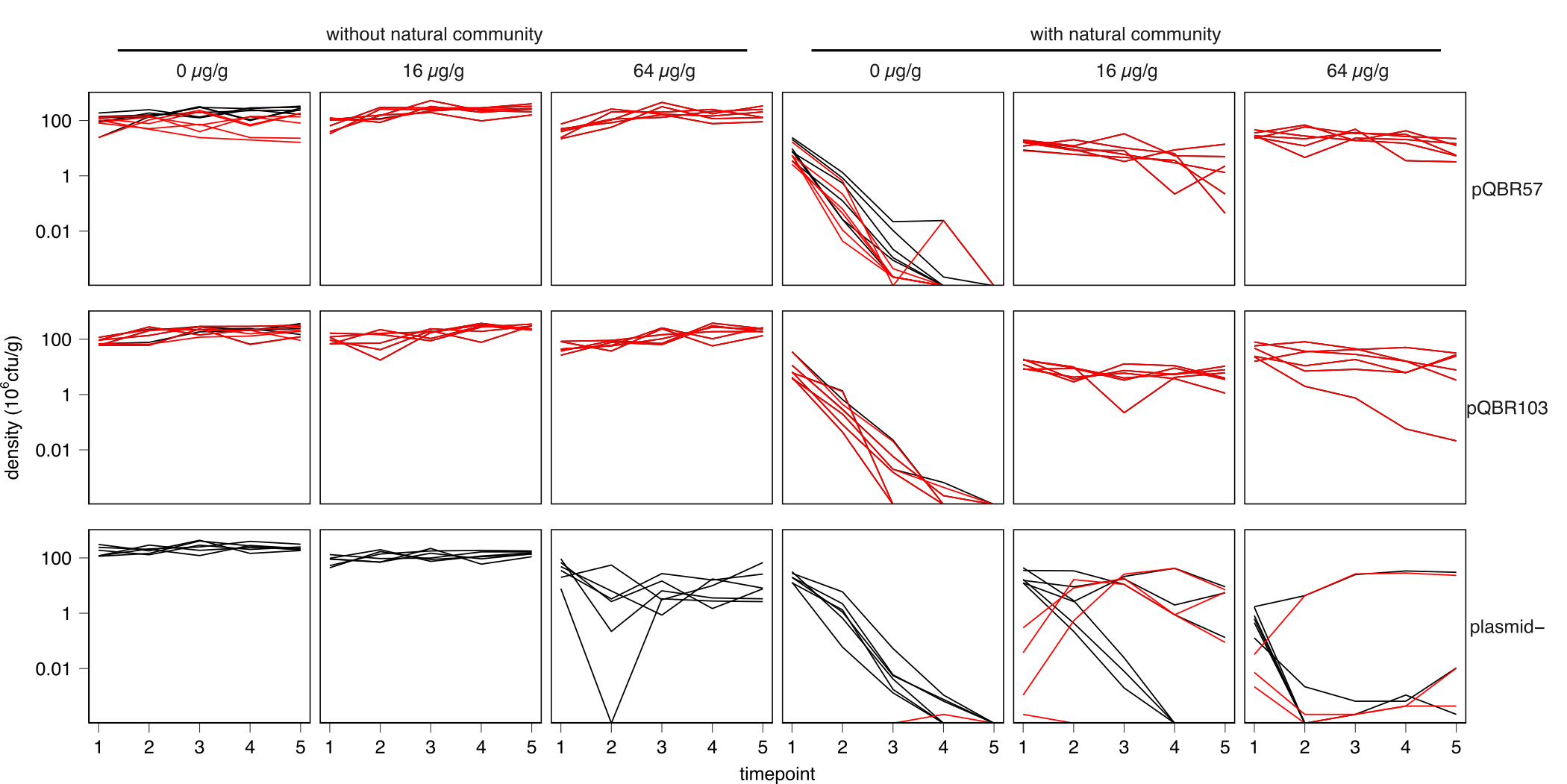

Supplement: FIGURE S1 — Mercury resistance dynamics in P. fluorescens SBW25 largely mirror the broader population dynamics. Lines in black are drawn according to Figure 1. Lines in red indicate dynamics of the mercury resistant compartments of the populations. Six replicate populations were established for each combination of treatments. [file Image_1.JPEG]

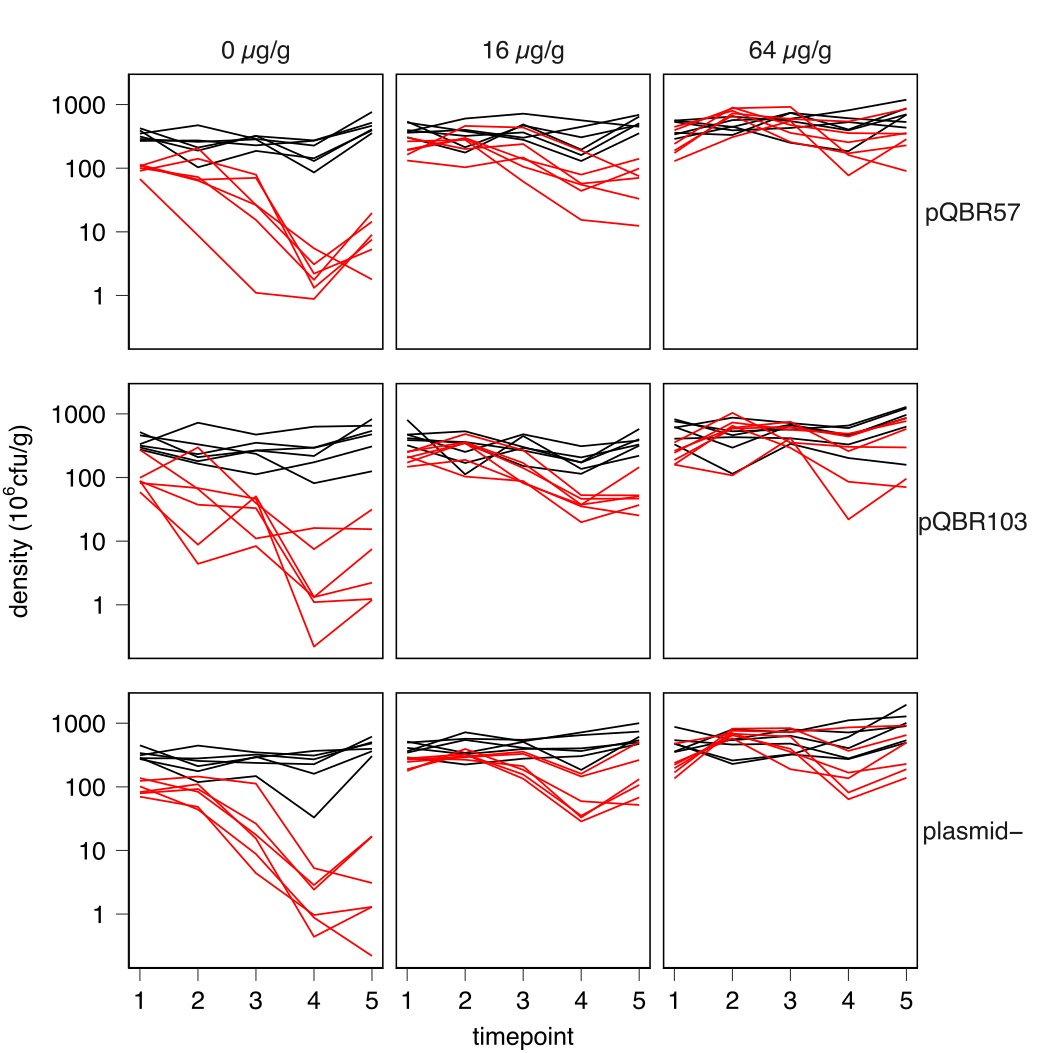

Supplement: FIGURE S2 — Population dynamics of the total community. Lines in black describe total population dynamics, while lines in red indicate dynamics of the mercury resistant compartments of the populations, as with Figure S1. Note that population dynamics were calculated from cfu grown on 0.1× nutrient agar and thus represents only part of the culturable heterotrophic portion of the community. Six replicate populations were established for each combination of treatments. [file Image_2.JPEG]

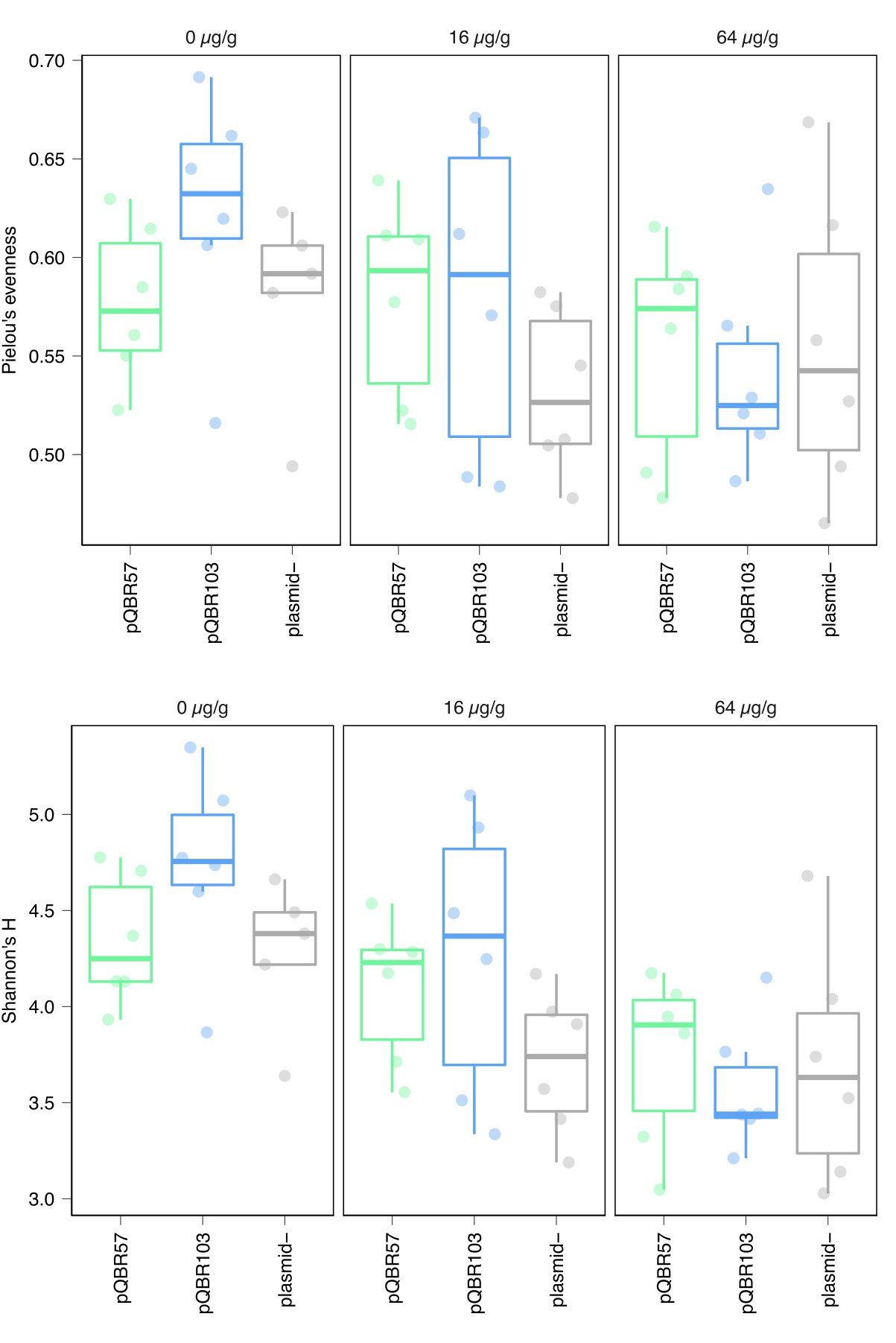

Supplement: FIGURE S3 — Effects of experimental treatments on alpha diversity (Shannon’s H, Pielou’s evenness). Figures are displayed as Figure 3. We detected a significant effect of mercury, but not plasmid, on Shannon’s H (effect of mercury, F2,48 = 11.8, p = 6.63e-5; effect of plasmid F2,48 = 1.7, p = 0.19). We did not detect significant effects of either treatment on Pielou’s evenness (effect of mercury, F2,48 = 2.5, p = 0.09; effect of plasmid F2,48 = 1.01, p = 0.37). [file Image_3.JPEG]

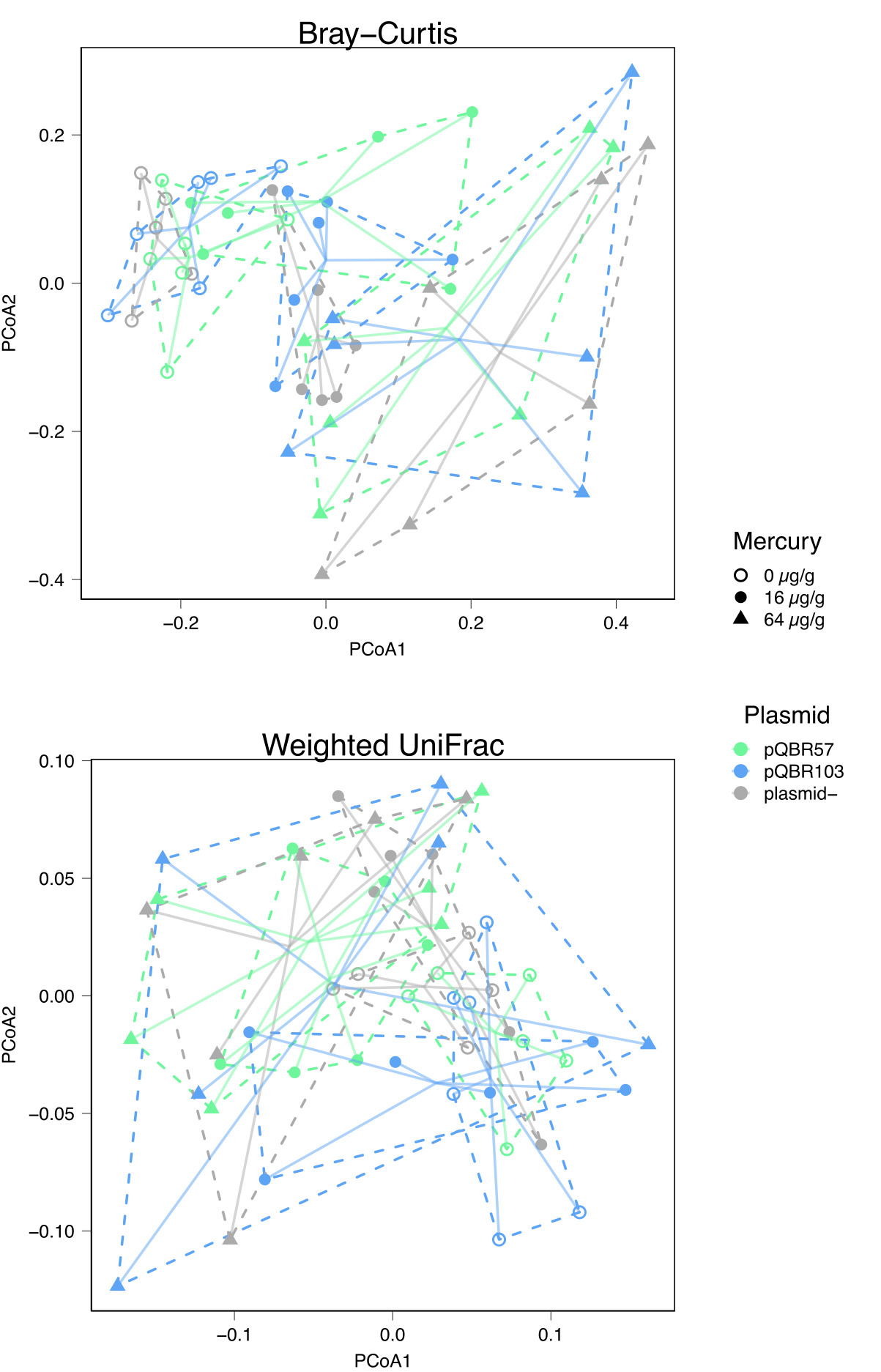

Supplement: FIGURE S4 — Effects of experimental treatments on community composition differences (Bray–Curtis distance, weighted UniFrac). Principal coordinates analysis of Bray–Curtis (top) and weighted UniFrac (bottom) distances. Plot is displayed as Figure 4. For Bray–Curtis, PCoA1 = 21.6% of the variance; PCoA2 = 11.8% of variance; effect of mercury pseudo-F = 6.13, p = 0.001; effect of plasmid pseudo-F = 0.85, p = 0.64. For weighted UniFrac, PCoA1 = 42.9% of the variance; PCoA2 = 16.7% of variance; effect of mercury pseudo-F = 5.23, p = 0.001; effect of plasmid pseudo-F = 0.99, p = 0.43. [file Image_4.JPEG]

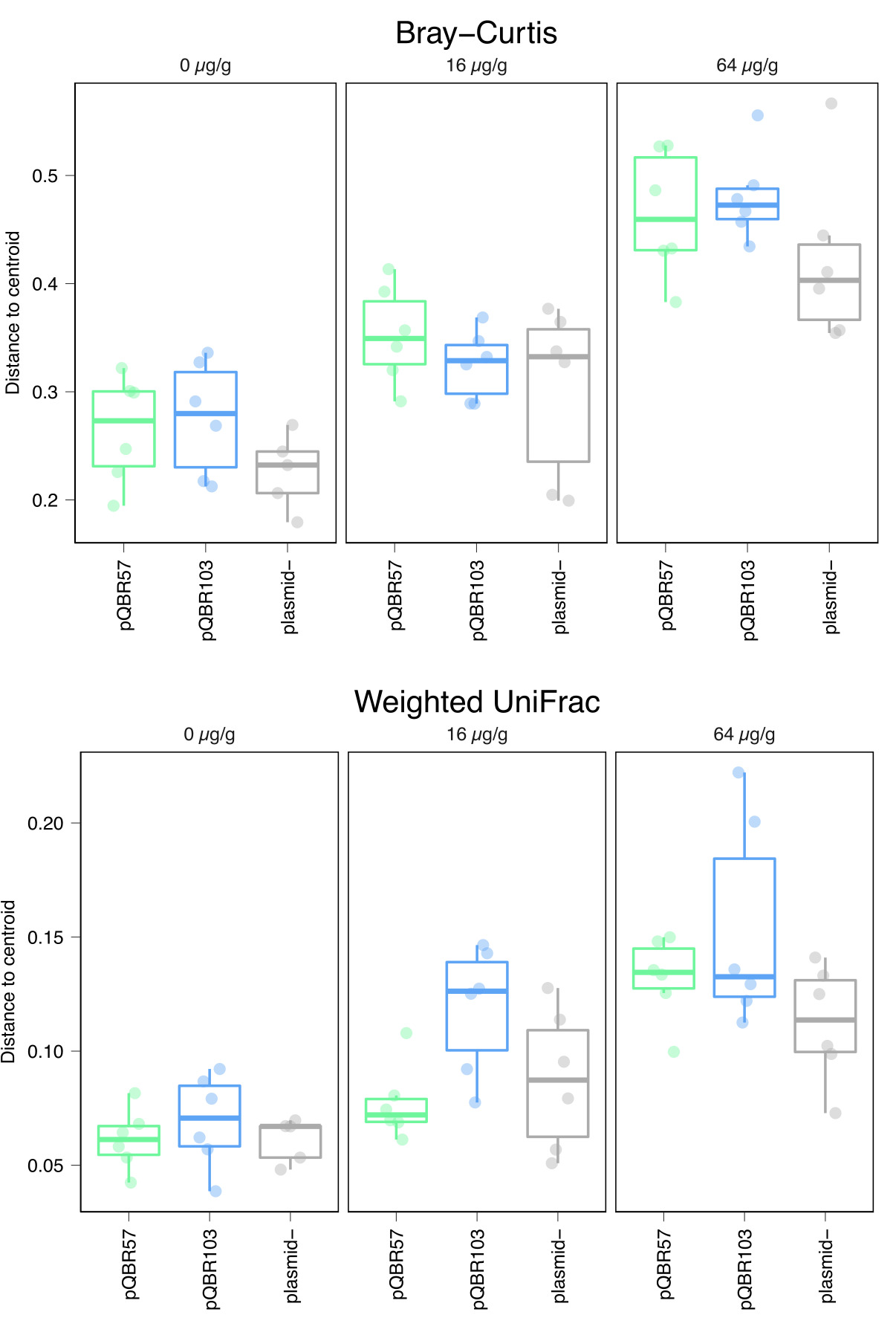

Supplement: FIGURE S5 — Effects of experimental treatments on community composition dispersion (Bray–Curtis distance, weighted UniFrac). Beta dispersion analysis of Bray–Curtis (top) and weighted UniFrac (bottom) distances. Plot is displayed as Figure 5. For Bray–Curtis, plasmid:mercury interaction F4,44 = 0.3, p = 0.8723. For weighted UniFrac, plasmid:mercury interaction F4,44 = 1.22, p = 0.32. [file Image_5.JPEG]

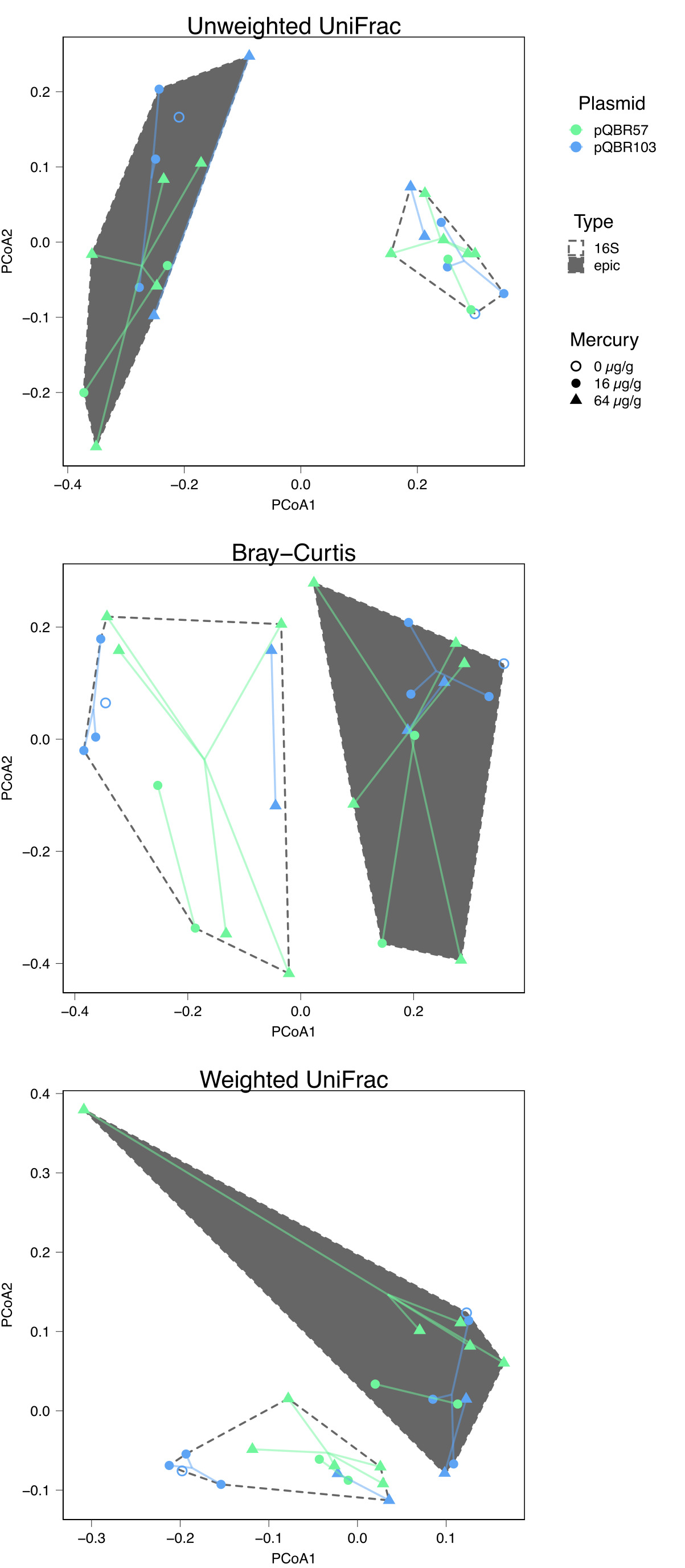

Supplement: FIGURE S6 — epicPCR samples a separate compartment of the community to general 16S amplicon sequencing. Principal coordinates analysis of unweighted UniFrac (top), Bray–Curtis (middle), and weighted UniFrac (bottom) distances, comparing epicPCR and whole-population 16S amplicon sequencing approaches. The amplicon corresponding to P. fluorescens SBW25 was removed to ensure that only the effects of merA transmission were analyzed. Each point indicates a sample, with colors and shapes indicating the treatment of the corresponding population (colors indicating different plasmid treatments, shapes indicating mercury treatments). Solid lines connect replicate treatments to the group centroid. Samples prepared with the same technique (epicPCR or 16S) are enclosed within dotted lines, and the area is shaded for the epicPCR samples for clarity. The variances explained by each axis for each distance are as follows: unweighted UniFrac PCoA1 = 47.2%, PCoA2 = 8.7%; Bray–Curtis PCoA1 = 16%, PCoA2 = 11.2%; Weighted UniFrac PCoA1 = 34.8%, PCoA2 = 24.3%. [file Image_6.JPEG]
