# Supplementary material for: The Impact of Mercury Selection and Conjugative Genetic Elements on Community Structure and Resistance Gene Transfer
Source: Front Microbiol. 2020 Aug 5;11:1846. doi: 10.3389/fmicb.2020.01846 (PMC7419628; doi:10.3389/fmicb.2020.01846)
Supplement: Supplementary file 7 [file Data_Sheet_1.docx]

**Supplementary Table 1.** Effects of experimental treatments on beta diversity assessed by weighted UniFrac. Permutation test for *adonis2* under reduced model, terms added sequentially (first to last).

|  | Df | SS | R^2^ | F | Pr(>F) |  |
| --- | --- | --- | --- | --- | --- | --- |
| Mercury | 2 | 0.13685 | 0.17304 | 5.1349 | **0.001** | *** |
| Plasmid | 2 | 0.02938 | 0.03715 | 1.1025 | 0.341 |  |
| Mercury:Plasmid | 4 | 0.03829 | 0.04841 | 0.7183 | 0.789 |  |
| Residual | 44 | 0.5863 | 0.74139 |  |  |  |
| Total | 52 | 0.79082 | 1 |  |  |  |

**Supplementary Table 2.** Effects of experimental treatments on beta diversity assessed by unweighted UniFrac. As Supplementary Table 1.

|  | Df | SS | R^2^ | F | Pr(>F) |  |
| --- | --- | --- | --- | --- | --- | --- |
| Mercury | 2 | 1.7126 | 0.44324 | 20.0995 | **0.001** | *** |
| Plasmid | 2 | 0.0923 | 0.0239 | 1.0838 | 0.327 |  |
| Mercury:Plasmid | 4 | 0.1843 | 0.0477 | 1.0815 | 0.332 |  |
| Residual | 44 | 1.8745 | 0.48516 |  |  |  |
| Total | 52 | 3.8638 | 1 |  |  |  |

**Supplementary Table 3.** Effects of experimental treatments on beta diversity assessed by Bray-Curtis dissimilarity. As Supplementary Table 1.

|  | Df | SS | R^2^ | F | Pr(>F) |  |
| --- | --- | --- | --- | --- | --- | --- |
| Mercury | 2 | 1.9378 | 0.19708 | 6.1439 | **0.001** | *** |
| Plasmid | 2 | 0.3184 | 0.03238 | 1.0095 | 0.433 |  |
| Mercury:Plasmid | 4 | 0.6377 | 0.06485 | 1.0109 | 0.428 |  |
| Residual | 44 | 6.9389 | 0.70569 |  |  |  |
| Total | 52 | 9.8329 | 1 |  |  |  |

**Supplementary Table 4.** Effects of experimental treatments on beta dispersion assessed by weighted UniFrac. ANOVA on distances to centroid extracted using *betadisper*, Type II Sums of Squares.

|  | SS | Df | F | Pr(>F) |  |
| --- | --- | --- | --- | --- | --- |
| Mercury | 0.042084 | 2 | 32.2521 | **2.38E-09** | *** |
| Plasmid | 0.007876 | 2 | 6.0356 | **0.005** | ** |
| Mercury:Plasmid | 0.003173 | 4 | 1.2158 | 0.318 |  |
| Residuals | 0.028707 | 44 |  |  |  |

**Supplementary Table 5.** Effects of experimental treatments on beta dispersion assessed by unweighted UniFrac. ANOVA on distances to centroid extracted using *betadisper*, Type II Sums of Squares.

|  | SS | Df | F | Pr(>F) |  |
| --- | --- | --- | --- | --- | --- |
| Mercury | 0.009878 | 2 | 9.6027 | **3.46E-04** | *** |
| Plasmid | 0.00029 | 2 | 0.2819 | 0.756 |  |
| Mercury:Plasmid | 0.0025236 | 4 | 1.2267 | 0.313 |  |
| Residuals | 0.0226307 | 44 |  |  |  |

**Supplementary Table 6.** Effects of experimental treatments on beta dispersion assessed by Bray-Curtis. ANOVA on distances to centroid extracted using *betadisper*, Type II Sums of Squares.

|  | SS | Df | F | Pr(>F) |  |
| --- | --- | --- | --- | --- | --- |
| Mercury | 0.36107 | 2 | 58.9086 | **3.61E-13** | *** |
| Plasmid | 0.02236 | 2 | 3.6484 | **0.034** | * |
| Mercury:Plasmid | 0.00375 | 4 | 0.3062 | 0.872 |  |
| Residuals | 0.13485 | 44 |  |  |  |

**Supplementary Table 7.** Primers used in this study.

| **Primer name** | **Primer sequence** | **Primer application** | **Reference** |
| --- | --- | --- | --- |
| merA_F1B | GTGTACTGTTGGATGAACG | First round epicPCR amplification | This study |
| merA_F2+R1 | GWATTACCGCGGCKGCTG GCGACCAGCTTGATGAAC |  | This study |
| R1 | GGYTACCTTGTTACGACTT |  | Spencer et al. 2016 |
| merA_F3E | ACACGACGCTCTTCCGATCTYRYR TGAGCCTGGACAACGTG | Second round epicPCR amplification | This study |
| PE16S_V4_E786_R | CGGCATTCCTGCTGAACCGCTCTTCCGATCT GGACTACHVGGGTWTCTAAT |  | Spencer et al. 2016 |
| R1+F1block10F | TTTTTTTTTT CAGCMGCCGCGGTAATWC 3SpC3 |  | This study |
| R1+F1block10R | TTTTTTTTTT GWATTACCGCGGCKGCTG 3SpC3 |  | This study |
| PE16S_V4_U515_F | ACACGACGCTCTTCCGATCTYRYR GTGCCAGCMGCCGCGGTAA | 16S amplicon sequencing | Spencer et al. 2016 |
| PE16S_V4_E786_R | CGGCATTCCTGCTGAACCGCTCTTCCGATCT GGACTACHVGGGTWTCTAAT |  | Spencer et al. 2016 |
