# Supplementary material for: The Impact of Mercury Selection and Conjugative Genetic Elements on Community Structure and Resistance Gene Transfer
Source: Front Microbiol. 2020 Aug 5;11:1846. doi: 10.3389/fmicb.2020.01846 (PMC7419628; doi:10.3389/fmicb.2020.01846)
Supplement: Supplementary file 8 [file Data_Sheet_2.docx]

# Supplementary Methods for ‘The impact of mercury selection and conjugative genetic elements on community structure and resistance gene transfer’

**James P. J. Hall^1,2,3^, Ellie Harrison^2^, Katariina Pärnänen^4^, Marko Virta^4^, Michael A. Brockhurst^2,5^**

^1^Department of Evolution, Ecology and Behaviour, Institute of Integrative Biology, University of Liverpool, Liverpool, L69 7ZB, UK

^2^Department of Animal and Plant Sciences, University of Sheffield, Sheffield, S10 2TN, UK

^3^Department of Biology, University of York, YO10 5DD

^4^Department of Microbiology, University of Helsinki, P.O.B 56, Helsinki, Finland

^5^Division of Evolution and Genomic Sciences, School of Biological Sciences, University of Manchester, Manchester, M13 9PT, UK

### Generating acrylamide beads for epicPCR

Un-lysed cells were used to generate acrylamide beads for epicPCR according to Spencer et al. (2016). A suspension of cells (approx. 10-20 million cells in 30 µl water) were mixed by gentle vortexing in a 2 ml round-bottom microcentrifuge tube with 200 µl acrylamide solution (12% acrylamide, 0.32% N-N’-bis(acryloyl)cystamine) and 25 µl amminium persulfate (10% w/v in water). STT emulsion oil (4.5% Span 80, 0.4% Tween 80, 0.05% Triton X-100 v/v in mineral oil) was added (600 µl) and the combined aqueous and oil phases were vortexed at maximum speed for 30 seconds. To polymerise the acrylamide, 25 µl tetramethylethylenediamine (TEMED) was added and the sample again vortexed at maximum speed for 30 seconds before incubating at room temperature for 90 minutes. To purify the acrylamide beads, 800 µl diethyl ether was added and the tube immediately mixed to generate a precipitate. The ether/oil mixture around the precipitate was removed, and the precipitate, which contained the beads, was washed five times in nuclease-free water. Washing was achieved by addition of 1 ml water, pelleting at 12 G for 30 seconds, and removing the water until all oil was removed. Remaining water was removed and beads were resuspended in 1 ml TK buffer (20 mM Tris-HCl pH 7.5, 60 mM KCl), and passed through a 35 µm cell strainer.

Cells within beads were lysed by treating 50 µl samples of beads with 0.4 µl Ready-Lyse Lysozyme (35 U/µl, epicentre, Madison, WI, USA) and incubating at 37°C overnight. Samples were centrifuged at 12 G for 30 seconds, the supernatant removed, and the pellet resuspended in 40 µl TK buffer. Proteinase K (10 µl, 1 mg/ml, Sigma P6556-5MG) and 0.4 µl Triton X-100 were added and incubated at 37°C for 30 minutes, followed by 95°C for 10 minutes. Beads were then washed 3 times in TK buffer and stored at 4°C until use.

Performing epicPCR reactions

epicPCR reactions were prepared by mixing a master mix consisting, for each sample, of 20 µl 5X Phusion HF buffer, 2 µl 50 mM MgCl_2_, 10 µl 10 µM merA_F1B primer, 10 µl 10µM R1 primer, 1 µl 1 µM merA_F2+R1 primer, 0.5 µl 20 mg/ml molecular biology grade BSA, 0.2 µl Tween 20, 8 µl Phusion Hot Start Flex polymerase (New England Biolabs M0535), 1 µl nuclease-free water. Samples of the master mix (55.2 µl) were mixed with 45 µl polyacrylamide beads by pipetting, and added to a 2 ml round-bottom tube containing four 2 mm sterile glass beads and 900 µl ABIL emusion oil (4% ABIL EM-90 (Evonik, Essen, Germany), 0.05% Triton X-100 v/v in mineral oil). Emulsions were generated by vortexing at maximum speed for 1 minute, and the reactions distributed across 16 PCR tubes (60 µl per tube). Reaction conditions were 1 min 94°C denaturation, followed by 33 cycles of 20 sec 94°C denaturation, 30 sec 52°C annealing, 45 sec 72°C extension, followed by a final extension at 72°C for 5 minutes. Immediately after completion, reactions were pooled and 2 µl 50 mM EDTA was added and stored at 4°C. Products were purified by centrifuging the reactions (13 G for 5 minutes) and the upper oil phase removed. Two extractions were performed with diethyl ether, by added 1 ml diethyl ether to each sample, mixing well by vortexing, centrifuging briefly, and discarding the upper phase. One extraction was performed with ethyl acetate, and then two further extractions performed with diethyl ether. Samples were left for remaining solvent to evaporate for approximately 10 minutes, and 100-150 µl sample was collected from the bottom phase. DNA was purified from the reactions using AMPure XP beads (Beckman Coulter, A63880), washed twice with 70% v/v ethanol, and eluted in 40 µl buffer EB (QIAGEN).

Second-round epicPCR products were generated using primers merA_F3E and PE16S_V4_E786_R. Blocking primers R1+F1block10F and R1+F1block10R were added to block amplification of unfused products. Reaction components were: 5 µl HF buffer 5x, 0.5 µl 10 µM dNTPs, 2.5 µl each amplification primer merA_F3E and PE16S_V4_E786_R (3 µM), 2.5 µl each blocking primer R1+F1block10F and R1+F1block10R (32 µM), 0.25 µl enzyme, 5 µl purified product from reaction 1, and 4.25 µl dH_2_O. Reaction conditions were 1 min 98°C denaturation, followed by 40 cycles of 20 sec 98°C denaturation, 30 sec 58°C annealing, 30 sec 72°C extension, followed by a final extension at 72°C for 5 minutes. Quadruplicate reactions were performed for each sample and the products pooled and purified using AMPure XP beads.

Full details of the epicPCR protocol including an instructional video can be found in Spencer et al. (2016).

### DNA extraction for 16S amplicon generation

Cells extracted using the nycodenz protocol were suspended in 25 µl TES and treated with 1 µl lysozyme (1250 U/ml) at 37°C for 30 minutes. TES (175 µl) and Triton X-100 (2 µl) were added, and the ‘Purification of total DNA from crude lysates using the DNeasy Blood & Tissue Kit’ (QIAGEN) was used to purify DNA, with a 30 minute incubation at 56°C following Buffer AL addition. DNA was eluted in 100 µl and 5 µl used for PCR using primers PE16S_V4_U515_F and PE16S_V4_E786_R. Reactions were performed using Phusion Hot-Start Flex. Reaction components were: 5 µl HF buffer 5x, 0.5 µl 10 µM dNTPs, 2.5 µl each primer (3 µM), 0.25 µl enzyme, 1 µl DNA template, and 13.25 µl H_2_O. Reaction conditions were 1 min 98°C denaturation, followed by 30 cycles of 20 sec 98°C, 30 sec 52°C, 30 sec 72°C, followed by a final extension at 72°C for 5 minutes. Quadruplicate reactions were performed for each sample and the products pooled.

### Addition of Illumina sequencing barcodes and sequencing of 16S and epicPCR amplicons

Illumina sequencing barcodes were added by PCR using the following reaction components: 5 µl 5x HF buffer, 0.5 µl 10 µM dNTPs, 1 µl each primer (10 µM), 0.25 µl Phusion Hot-Start Flex polymerase, 13.25 µl dH_2_O, 4 µl purified product (either epicPCR reaction 2, or 16S amplification product). Reaction conditions were 1 min 98°C denaturation followed by 7 cycles of 30 sec 98°C, 30 sec 83°C, 30 sec 72°C, followed by 5 min 72°C final extension. Quadruplicate reactions were performed for each sample and the products pooled.

Products were run on an agarose gel to assess purity and concentration. 16S samples were pooled by mixing 5 µl of each barcoded sample. EpicPCR samples were pooled by mixing 5 µl of each barcoded sample that yielded a clear band on the gel, and 10 µl from each sample that did not produce a clear band (likely to due low/no yield). Each library was sequenced using a MiSeq v2 with 250 bp paired-end reads. The 16S amplicon analyses generated >50,000 read pairs per sample library. Yield from epicPCR was variable due to low input from some samples.

### References

Spencer, S.J., Tamminen, M.V., Preheim, S.P., Guo, M.T., Briggs, A.W., Brito, I.L., et al. (2016) Massively parallel sequencing of single cells by epicPCR links functional genes with phylogenetic markers. *ISME J.* **10**: 427–436.
